# Survival after Stereotactic Radiosurgery in the Era of Targeted Therapy: Number of Metastases No Longer Matters

**DOI:** 10.3390/curroncol31060228

**Published:** 2024-05-28

**Authors:** James de Boisanger, Martin Brewer, Matthew W. Fittall, Amina Tran, Karen Thomas, Sabine Dreibe, Antonia Creak, Francesca Solda, Jessica Konadu, Helen Taylor, Frank Saran, Liam Welsh, Nicola Rosenfelder

**Affiliations:** 1 The Royal Marsden Hospital, London SW3 6JJ, UK; 2 The Institute of Cancer Research, London SM2 5NG, UK; 3 Cancer Institute, University College London, London WC1E 6BT, UK; 4 Cancer and Blood Service, Auckland City Hospital, Auckland 1023, New Zealand

**Keywords:** stereotactic radiosurgery, brain metastases, number of metastases

## Abstract

Randomised control trial data support the use of stereotactic radiosurgery (SRS) in up to 4 brain metastases (BMs), with non-randomised prospective data complementing this for up to 10 BMs. There is debate in the neuro-oncology community as to the appropriateness of SRS in patients with >10 BMs. We present data from a large single-centre cohort, reporting survival in those with >10 BMs and in a >20 BMs subgroup. A total of 1181 patients receiving SRS for BMs were included. Data were collected prospectively from the time of SRS referral. Kaplan–Meier graphs and logrank tests were used to compare survival between groups. Multivariate analysis was performed using the Cox proportional hazards model to account for differences in group characteristics. Median survival with 1 BM (*n* = 379), 2–4 BMs (*n* = 438), 5–10 BMs (*n* = 236), and >10 BMs (*n* = 128) was 12.49, 10.22, 10.68, and 10.09 months, respectively. Using 2–4 BMs as the reference group, survival was not significantly different in those with >10 BMs in either our univariable (*p* = 0.6882) or multivariable analysis (*p* = 0.0564). In our subgroup analyses, median survival for those with >20 BMs was comparable to those with 2–4 BMs (10.09 vs. 10.22 months, *p* = 0.3558). This study contributes a large dataset to the existing literature on SRS for those with multi-metastases and supports growing evidence that those with >10 BMs should be considered for SRS.

## 1. Introduction

The number of people diagnosed and living with brain metastases (BMs) has increased over time [1] due to improved survival with cancer [2] allowing more time for BM development either as a sanctuary site or with synchronous extracranial relapse and due to increased MRI brain surveillance resulting in earlier detection of asymptomatic disease [3]. At the same time, the prognosis of patients with BMs is improving [3] due to better systemic treatments for extracranial control and better targeted intracranial treatment with stereotactic radiosurgery (SRS). Many newer systemic therapies penetrate the brain with good intracranial activity [4,5,6,7,8], such that combining and sequencing multi-modal BM treatment is becoming a key area of interest [9], with at least 15 trials currently in progress combining SRS with immune checkpoint inhibitors [10]. In the 1970s, median survival after the development of BMs was 3–4 months with whole brain radiotherapy (WBRT) [11] (with a significant risk of neuro-cognitive decline and a reduction in quality of life) [12,13], rising to 6 months at the time of the first SRS randomised control trial in 2004 [14] and to over 10 months a decade later [13,15], with many patients now living for years after diagnosis of BMs [16]. 

All randomised controlled trials assessing the benefit of SRS have been performed in patients with 4 BMs or fewer [12,13,17,18], with the greatest benefit observed in those with 1 BM [14]. Andrews et al. demonstrated improved survival with the addition of SRS to WBRT in those with 1 BM and improved local control rates for patients with 1–3 BMs [14]. Aoyama et al. and Kocher et al. showed no improvement in survival when WBRT was added to SRS alone in the treatment of 3 (Kocher) and 4 (Aoyoma) BMs [17,18]. Chang et al. and Brown et al. reported worse cognitive outcomes in patients with 1–3 BMs treated with SRS plus WBRT vs. those treated with SRS alone [13], with Chang et al. also demonstrating worse actuarial survival outcomes [12]. 

Evidence for the use of SRS in those with >4 BMs is limited to prospective or retrospective evaluations of outcomes in cohort series. Despite this, the use of SRS in patients with 5–10 BMs has become widespread such that a randomised trial comparing SRS with WBRT in those with 4–10 BMs failed to recruit [19]. 

There have been conflicting results from studies as to whether survival reduces as the number of BMs increases. Data from a large (1194 patients) non-randomised prospective study in 2014 showed similar outcomes in those with 2–4 BMs (*n* = 531) and 5–10 BMs (*n* = 208) [20]. The investigators reported a median survival of 10.8 months in both groups (HR 0.97, 95% CI 0.81–1.18, *p* = 0.78). A retrospective cohort study of 2553 patients showed similar overall survival (OS) for those with >/=10 BMs to those with 2–9 BMs (OS 6.8 months vs. 6.0 months, *p* = 0.10) [21], and a further retrospective analysis of >2000 patients did not demonstrate a significant difference in survival between those with 2–4 and 5–15 metastases (OS 9.5 m vs. 7.5 m, *p* = not significant) [22]. Interestingly, there was no difference in survival between patients with non-small-cell lung cancer with >20 BMs and those with a solitary BM treated with SRS (OS 15 m vs. 12 m, *p* = 0.3) in a small sample of patients when matched for disease volume (*n* = 82) [23]. However, data on driver mutational status were not reported between groups. 

Conversely, a large retrospective study of >5700 patients demonstrated a statistically significant difference in OS between patients with 5–10 BMs and >10 BMs (OS 6.3 m vs. 5.5 m, *p* = 0.025) and 2–10 BMs and >10 BMs in modified collapsed number of metastases groupings (OS 6.4 m vs. 5.5 m, *p* ≤ 0.001) [24]. This finding was supported by a further retrospective cohort study (based on a prospectively accumulated database) of >2000 patients showing a significant survival difference in those with 5–10 BMs and 11–20 BMs (OS 7.7 m vs. 6.5 m, *p* = 0.0001) [25]. A more recent systematic review and meta-analysis, which included 15 papers from 822 screened, also showed statistically worse OS in patients with ≥10 BMs compared to 10 BMs (HR 1.10, *p* ≤ 0.01) [26]. 

It is notable that in all these series, survival in those with >10 BMs is clinically comparable to the control arm and closely approaches or exceeds 6 months, even in studies demonstrating statistically significant survival differences with an increasing number of BMs. 

Whilst many in the neuro-oncology community believe that SRS has a role for patients with >10 BMs, reflected in treatment guidelines which have removed the tumour number criterion for treatment (e.g., the guidelines of the National Comprehensive Cancer Network and Congress of Neurological Surgeons) [27,28], there is ongoing debate, and other guidelines continue to distinguish between those with ≤4 BMs and those with >4 BMs (e.g., the guidelines of the American Society of Clinical Oncology, the American Society of Neuro-oncology, the American Society for Radiation Oncology) [4]. This may be due to the concern of ‘mission creep’, in that the comparison arms (e.g., 2–10 BMs) should not be considered the standard arm as the randomised trials were limited to ≤4 BMs, and due to concern that there is more likely to be disseminated disease in those with >10 BMs, negating the rationale for a highly localised treatment such as SRS. 

This study aims to address the ongoing debate as to the appropriateness of SRS in patients with >10 BMs in the absence of randomised controlled trial data and compares the outcomes in those with 1, 2–4, 5–10, and >10 BMs (with further data assessing outcomes in those with >20 BMs presented in additional analyses). We present the results from a large prospectively collected single-centre cohort of patients, treated according to a standard set of national commissioning criteria [29] in a uniform manner over a 7-year period from 2016 to 2023 and seek to complement existing research evaluating the impact of the number of BMs on survival. 

## 2. Materials and Methods

A total of 1181 patients were included in the study. Patients with BMs treated with SRS at our institution between April 2016 and January 2023 were included. Patients were treated according to UK national commissioning guidelines [29] and required to meet the following National Health Service (NHS) criteria: Karnofsky Performace Score (KPS) >70, absent, controlled or controllable extracranial disease, total tumour volume ≤20 cc, and life expectancy estimated at >6 months. SRS was delivered using Cyberknife or Linac radiotherapy platforms and patients received single- and/or multi-fraction SRS. Patients who received SRS multiple times were only included once, and the data reported are only from the first course of SRS. Those who had received WBRT or neurosurgery prior to first SRS were eligible for inclusion. 

Our hospital is part of a neuro-oncology network covering South London, Surrey, and Sussex (contiguously located regions of Southern England, UK), with a catchment area population estimated at 5 million people across rural and urban settings. Patients are referred by primary oncology teams to the network SRS multi-disciplinary team meeting (MDT), attended by neurosurgeons, clinical oncologists, neuro-pathologists, neuro-radiologists, neurologists, and clinical nurse specialists. Those meeting the UK national commissioning criteria (outlined above) are accepted for consideration of SRS, which is then delivered at our centre. The system is designed to allow all patients in the catchment population access to specialist neuro-oncology treatments.

Patient demographic and treatment data were collected prospectively and consecutively at the time of SRS referral to create a large database suitable for retrospective analysis, thereby producing a ‘complete’ data set of all patients treated at our institution, reducing ascertainment bias and increasing real-world applicability. Radiotherapy treatment-related data were extracted from radiotherapy planning systems, and survival data were retrieved from the NHS Spine database. 

Survival was calculated from the date of first SRS treatment to allow for direct comparison with other studies. The Spine database was last accessed on 31 July 2023. A censoring date was chosen 3 months before this (31 April 2023) for those without a date of death to allow for delays in reporting. 

Data were analysed using Graphpad Prism software (v10.1.0). Kaplan–Meier graphs were produced to demonstrate differences in survival between groups, and logrank tests were conducted to compare the generated curves. Multivariable analysis was performed using the Cox proportional hazards model to account for differences in the clinical tumour characteristics between groups. BM number groups were pre-planned to provide the best match with the existing literature (see Introduction), and analyses were pre-specified. For multivariable analysis, age, gender, Karnofsky performance status (KPS), histology, molecular subtype, and total intracranial disease volume were analysed—each having been incorporated into published BM prognostic tools [16,30,31,32,33]. Continuous variables such as age and total intracranial tumour volume were categorised according to groups used in prognostic scoring systems. Likelihood ratio tests were conducted, comparing models in which each predictor variable was individually excluded, thus providing a measure of the contribution of each variable to survival (expressed as *p*-values). 

## 3. Results

Table 1 provides an overview of the demographics and descriptive variables of all patients. Median follow up was 38.65 months. The majority (89.16%) of patients had 10 or fewer metastases. The age and sex distribution were similar across groups defined by brain metastases number (1, 2–4, 5–10, >10) and differences between the groups were accounted for in the multivariable analysis. The proportion of patients with KPS 100 in the >10 BMs group (23.44%) was higher than the overall proportion with KPS 100 (17.02%). In terms of intracranial disease volume, the >10 BMs group had the smallest proportion, with <1.5 cc (17.19%), and the proportion of patients with >8.5 cc was higher than the overall proportion with >8.5 cc (22.66% vs. 20.66%).

The most common primary tumour histologies were lung (43.27%), breast (21.93%), and melanoma (13.80%) (Table 1). Using breast as the reference, there was no statistically significant difference in survival between histologies, apart from two notable exceptions, namely lung and gastrointestinal (GI) (*p* = 0.0021 and *p* ≤ 0.0001, respectively) (Table 2). We examined four molecular subtypes in greater detail, ALK, EGFR, BRAF, and HER2, as these mutations were actionable at the time of the study population’s treatment. These were present in almost one-third of patients overall (29.47%). A higher proportion of patients with >10 BMs had such driver mutations (49.22%) compared to the overall proportion with any driver mutation (29.47%). 

### 3.1. Impact of BM Number on Survival

**Figure 1:**
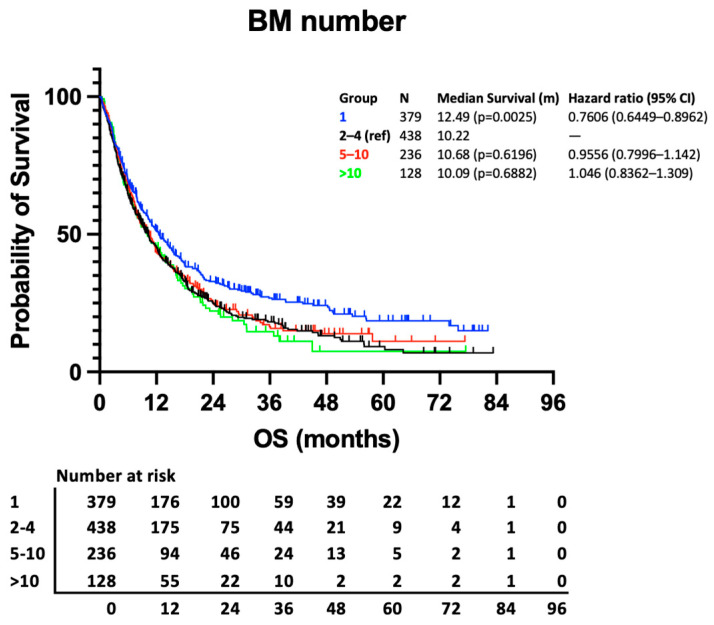
Kaplan–Meier curve presenting survival according to no. of BM groups.

Median survival is similar between all patient groups with more than 1 BM (Figure 1). Whilst the difference in survival between those with 1 BM and those with 2–4 BMs reaches statistical significance (12.49 vs. 10.22 months, *p* = 0.0025), the difference between 2–4 BMs and 5–10 BMs or >10 BMs is not statistically significant based on univariable analysis (10.22 vs. 10.68 months, *p* = 0.6196; and vs. 10.09 months, *p* = 0.6882, respectively). 

**Figure 2:**
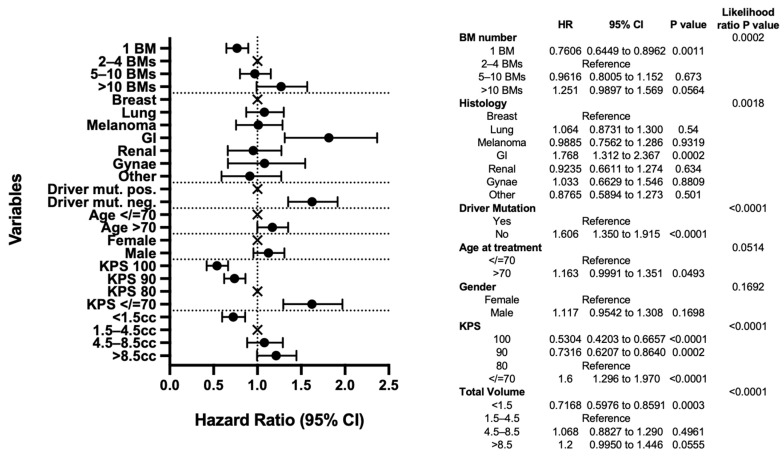
Multivariable analysis. Forest plot and table presenting the variables included in the analysis and their intra-group pair comparisons and inter-group likelihood ratio *p*-value results.

As expected, the results of our multivariable analysis demonstrate the significance of previously described prognostic factors including KPS, histology, molecular subtype, and total intracranial disease volume and support the univariable analysis. The improved survival of those with 1 BM remains significant when compared to those with 2–4 BMs (*p* = 0.0011), and here, the hazard ratio (HR) for death of those with >10 BMs, compared to those with 2–4 BMs, does not reach statistical significance (1.251, 95% CI 0.9897–1.569, *p* = 0.0564) (Figure 2). 

Also of note in the multivariable analysis, GI histology remains a significant predictor of poor outcomes (HR 1.768, 95% CI 1.312–2.367, *p* = 0.0002). However, lung cancer no longer reaches statistical significance (HR 1.064, 95% CI 0.8731–1.300, *p* = 0.54) when other factors, notably molecular status, are taken into account (Figure 2). 

The likelihood ratio test results reveal driver mutation status, KPS, and total tumour volume to be the most important predictors of survival (each *p <* 0.0001). When looking at the intra-group pair comparisons, total tumour volume <1.5 cc is particularly significant when compared with the 1.5–4.5 cc reference group (*p* = 0.003), as are KPS 100, 90, and <70 compared to KPS 80 reference (*p <* 0.0001, *p* = 0.0002, *p <* 0.0001, respectively). 

### 3.2. Impact of Histology and Molecular Subtype on Survival

**Figure 3:**
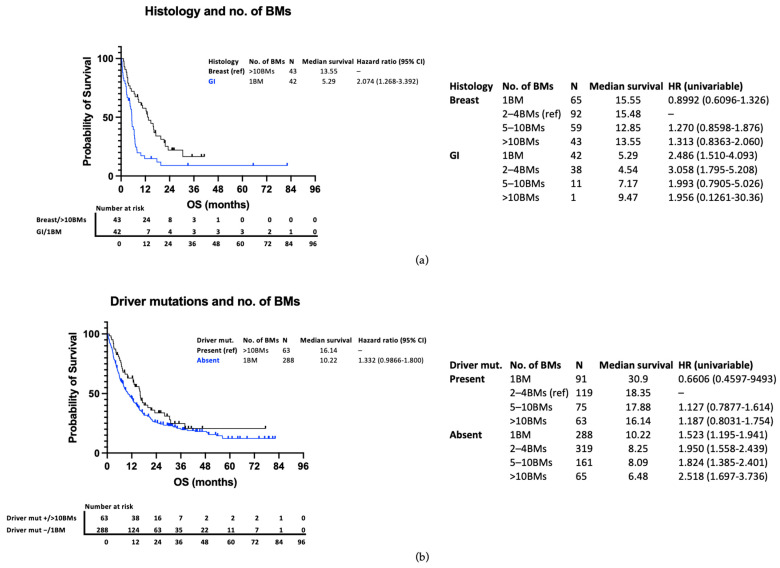
(**a**) Kaplan–Meier curve presenting survival in those with breast cancer and >10 BMs compared to those with a GI primary and a solitary BM. (**b**) Kaplan–Meier curve presenting survival in those with a driver mutation and >10 BMs compared to those without a driver mutation and a solitary BM.

**Figure 4:**
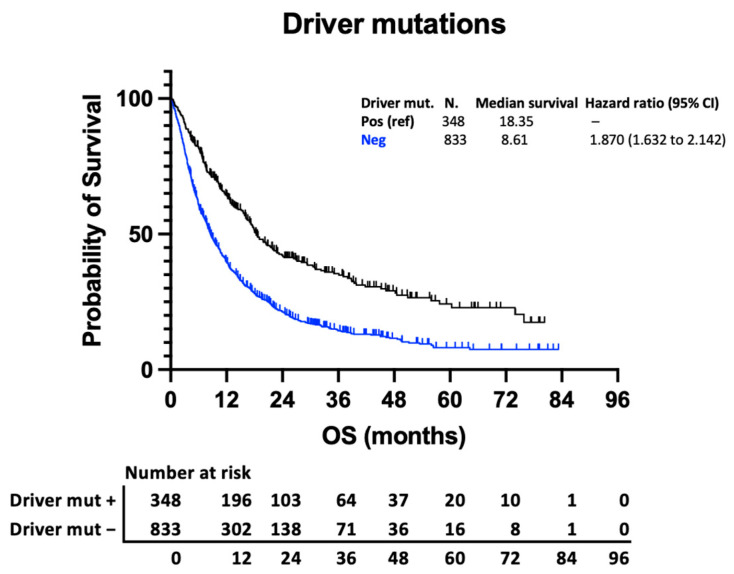
Kaplan–Meier curve demonstrating survival of those with targetable molecular alterations (EGFR, ALK, BRAF, and HER2) and those without.

Variations in survival within other prognostic subgroups are considerably greater than variation from the number of BMs. This is illustrated in Figure 3a, comparing outcomes of patients with more (breast) and less (GI) favourable histology, and Figure 3b, comparing outcomes of those with and without driver mutations. To demonstrate the point clearly, the outcomes of those with 1 BM in the less favourable group are compared with those with >10 BMs in the more favourable group. Patients with breast cancer and >10 BMs have a higher median survival than those with GI cancer and a solitary BM (13.55 vs. 5.29 months) (Figure 3a). Similarly, those with an actionable driver mutation have a survival advantage compared to those without (18.35 vs. 8.61 months) (Figure 4). When subdivided by number of metastases, there is a stepwise reduction in survival from those with 1 BM with a driver mutation to those with >10 BMs without a driver mutation (Figure 3b), with a clinically relevant longer survival in those with >10 BMs and a driver mutation than those with 1 BM and no driver mutation (16.14 vs. 10.22 months). 

### 3.3. Patients with >20 BMs Have Similar Survival to Those with 2–4 BMs

**Figure 5:**
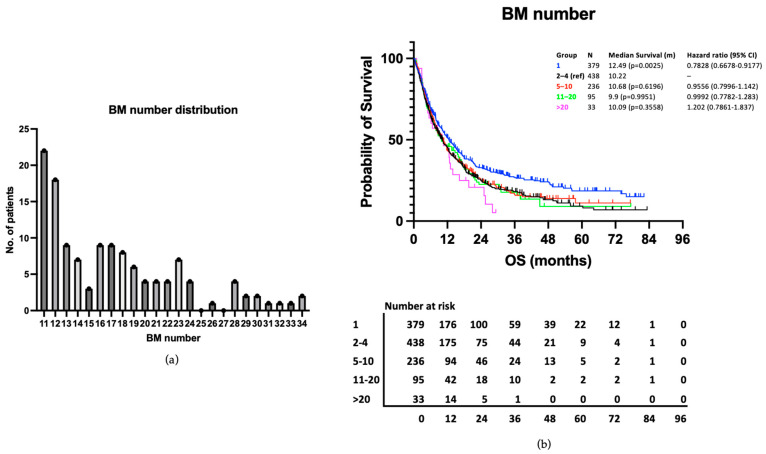
(**a**) Range and distribution of BM number in the >10 BMs group. (**b**) Kaplan–Meier curve presenting survival according to no. of BM groups, including the >20 BMs subgroup.

The distribution of BMs in the >10 BMs group (range 11–34) are presented in Figure 5a. As would be expected, this shows the majority (95/128, 74.22%) have 11–20 BMs, with a smaller number having >20 BMs (33/128, 25.78%). The survival outcomes of those with >20 BMs were examined as a subsection of the >10 BMs group. Median survival was 10.09 months compared to 10.22 months in the 2–4 BMs reference group and 10.68 months in the 5–10 BMs group. The difference in survival between the >20 BMs group and the 2–4 BMs reference group was not statistically significant in our univariable analysis (*p* = 0.3558) (Figure 5b). This patient group was not analysed separately in the multivariable analysis due to its small size.

## 4. Discussion

This study adds significantly to the existing literature by contributing a large dataset of patients undergoing SRS for >10 BMs. Our data show that those with a solitary brain metastasis can usually expect to live longer than those with >1 metastasis. This reinforces previous research which demonstrates this relationship [14,20], supporting our dataset as representative of the wider SRS population. When those with 2–4 BMs were compared to those with >10 BMs, we found no statistically significant difference in their survival in univariable (10.22 vs. 10.09 months, *p* = 0.6882) or multivariable (*p* = 0.0564) analyses. This is a similar result to the landmark 2014 retrospective analysis by Yamamoto et al., which reported survival values of 6.8 months in patients with 2–9 BMs and 6.0 months in those with ≥10 BMs [21]—a difference that was not statistically significant (HR 1.11, 95% CI 0.97–1.32, *p* = 0.10) nor clinically meaningful. We intentionally chose 2–4 BMs as our comparator group rather than 2–9 BMs to ensure we aligned our groupings most closely with those supported by randomised controlled trials (i.e., 1–4 BMs) and to avoid ‘statistical creep’ by including groups not supported by clinical trial data (i.e., 5–9 BMs) in our comparator arm.

The 2014 Yamamoto paper, despite apparently incorporating a dataset that was significantly larger than our own (2553 patients), actually included 720 patients in the comparative analysis of the two groups of interest (2–9 BMs and ≥10 BMs). The patients were equally split between the two groups (360 in each) using propensity score matching to create case-matched cohorts [21]. This compares to the 566 unmatched patients split unevenly between the 2–4 BMs (*n* = 438) and >10 BMs (*n* = 128) groups in our study. Instead of adopting a case-matched approach, we conducted multivariable analysis to account for differences in clinical factors between the groups. The variables included in our analysis showed considerable overlap with those included in the case matching of the Yamamoto paper (age, sex, primary tumour site, KPS, tumour volume). Yamamoto et al. also included extracerebral metastases, neurological symptoms, prior intracranial procedures, and peripheral doses, whereas we also included presence of driver mutations.

As mentioned in the Introduction, other large retrospective datasets found statistically significant differences in survival between >10 and ≤10 BM groups [24,25]. However, the absolute difference in median survival ranged from only 0.8 to 1.2 months in these studies. When combined with our study (absolute difference 0.13 months) and the 2014 Yamamoto paper (absolute difference 0.8 months), it is noticeable that the differences are small. Regardless of statistical significance, such differences are clinically insignificant. The median survival figures reported in this study are substantially higher in all groups than those from selected past publications [22,24,25] (i.e., ~4-month increase compared to the 2014 Yamamoto et al. paper of 10 years ago), and the small differences in survival mentioned above are even less significant when contextualised against a larger general increase in median survival.

This study is noteworthy among the large (>1000 patient) studies in that it subdivided the >10 BMs group into a >20 BMs subgroup. Survival in the >20 BMs group (10.09 m) was comparable with the 2–4 BMs (10.22 BMs) and 5–10 BMs groups (10.68 m). The inclusion of a separate >20 BMs group sought to provide more nuance in our understanding of the treatment of patients with multiple metastases and to assist the likely further debate around an absolute upper limit for SRS. The largest published study, considering 5000 patients, quantified a 4% increase in the hazard ratio of death for every additional 6–7 BMs [24]. This suggests there may be a ‘maximum’ number of BMs, above which the benefit of SRS, either in absolute or relative terms, becomes less clear. More metastases increase planning and treatment time. Evidence for a maximum number above which there is no benefit from SRS would be welcome, as it could add clinical support to difficult resource allocation decisions, particularly in resource-constrained publicly funded health systems. Furthermore, it could reconcile those who believe that localised treatments such as SRS are of dubious theoretical use in widespread intracranial disease by validating their concerns in those who are truly poly-metastatic. Unfortunately, our dataset, despite having >1000 patients, is too small to investigate subgroups beyond 20 BMs. We therefore feel that ‘how many is too many?’ will be the next pertinent question in the understanding of multi-metastatic treatment.

A related prognostic variable, brain metastasis velocity (BMV) (i.e., the rate of development of new BMs), may play a role in helping to answer this question. This variable was described by Farris et al. in 2017 as a way of predicting outcomes after initial distant brain failure (DBF) following SRS [34]. In this paper, BMV was calculated as the cumulative number of new BMs since initial SRS divided by the total time between initial SRS and DBF. The study found that higher BMV was associated with worse survival in both univariable and multivariable analyses, as well as increased rates of salvage WBRT and neurologic death. Interestingly, >2 BMs at presentation was predictive of higher BMV at DBF (*p* = 0.004), and, as a continuous variable, an increasing number of BMs at initial SRS was associated with increasing BMV (*p <* 0.001). This finding was supported by Hughes et al., who found median BMVs at the time of DBF of 3.9, 6.1, and 11.7 in patients with 1, 2–4, and 5–10 BMs, respectively (*p <* 0.01). Farris et al. suggested BMV as a useful prognostic metric in patients with progressive intracranial disease after SRS. However, BMV has not yet been applied to an intracranial-treatment-naïve BM population and may not be useful in this context unless a group can be identified where an active surveillance approach is appropriate.

Regarding our dataset, we recognise that survival was significantly lower (5.13 months) for those with GI cancer compared to other primaries. This finding is echoed in other published literature, with survival ranging from 3 to 9 months [35,36,37]. The largest of these was a population-based study using the Surveillance, Epidemiology, and End Results database of the National Cancer Institute, which included 28,736 patients with gastric adenocarcinoma. The authors report a median survival of 3 months and a 6-month cumulative mortality of 57% in those with brain metastases [35]. This is particularly relevant in the UK context, where national commissioning criteria impose a minimum expected survival of 6 months [29]. All patients treated in this series were expected to live for 6 months after SRS, as estimated by their referring oncology teams. Further work is needed to better understand those patients with GI cancers in whom SRS is truly appropriate.

Although the primary focus of this paper has been on the impact of number of intracranial metastases, the control of the systemic disease is likely to be the most important factor for survival in many cases. We use examples to demonstrate that primary histology (where BMs are a late feature of the disease and when few systemic options are available) or actionable molecular driver mutations can have considerably greater implications for survival than the number of BMs, yet these are not debated as exclusion criteria for SRS. Our study suggests that those with favourable histology and many BMs live longer than those with unfavourable histology and just 1 BM, and those with driver mutations live longer than those without, irrespective of the number of metastases. At the time of writing in 2024, patients without any targetable driver mutation and a single BM would likely be candidates for SRS in most centres, provided they met other treatment criteria. The median survival for this group in our study was 10.22 months. However, it is significant that in those with a targetable driver mutation and >10 BMs, the median survival was 16.14 months and remained above 6 months (6.48 months) even in those without a targetable driver mutation. On the basis of the data presented, the number of metastases should not be a barrier to SRS.

## 5. Conclusions

This study adds to the growing body of literature presenting survival in patients with >10 BMs. Our results suggest no significant survival difference between those with 2–4 metastases and those with >10 BMs in univariable or multivariable analysis in our ‘real-world’ prospectively collected data. Patients with >20 metastases had a median survival of 10.09 months, similar in absolute terms to those with 2–4 and 5–10 BMs and exceeding the 6-month minimum expected survival in UK national commissioning criteria [29]. Other prognostic factors significantly affect survival and play a clinically more important role in determining prognosis than the number of metastases. Our data indicate that patients with >10 BMs should not be refused SRS based on BM number alone. Further research is needed, likely requiring very large datasets, in order to investigate the question of whether there is a maximum number of BMs above which there is no survival benefit with SRS in different subpopulations of patients.

## Figures and Tables

**Table 1:** Demographic and descriptive variables.

Number of BMs	—	1	2–4	5–10	>10	Total	Proportion
Total		379 (32.09%)	438 (37.09%)	236 (19.98%)	128 (10.84%)	1181	100%
Deaths		269 (70.98%)	339 (77.40%)	184 (77.97%)	102 (79.69%)	894	75.70%
Median follow-up (m)		42.34	38.65	43.62	35.14	-	-
Age	≤70	265 (69.92%)	312 (71.23%)	177 (75%)	99 (77.34%)	853	72.23%
	>70	114 (30.08%)	126 (28.77%)	59 (25%)	29 (22.66%)	328	27.77%
Gender	Male	178 (46.97%)	187 (42.69%)	89 (37.71%)	43 (33.59%)	497	42.08%
	Female	201 (53.03%)	251 (57.31%)	147 (62.29%)	85 (66.41%)	684	57.92%
KPS	100	65 (17.15%)	70 (15.98%)	36 (15.25%)	30 (23.44%)	201	17.02%
	90	164 (43.27%)	210 (47.95%)	110 (46.61%)	51 (39.84%)	535	45.30%
	80	98 (25.86%)	94 (21.46%)	64 (27.12%)	34 (26.56%)	290	24.56%
	70	52 (13.72%)	63 (14.38%)	26 (11.02%)	13 (10.16%)	154	13.04%
	60	0 (0%)	1 (0.23%)	0 (0%)	0 (0%)	1	0.08%
Disease volume	<1.5 cc	143 (37.73%)	141 (32.19%)	59 (25.00%)	22 (17.19%)	365	30.91%
	1.5–4.5 cc	110 (29.02%)	107 (24.43%)	70 (29.66%)	55 (42.97%)	342	28.96%
	4.5–8.5 cc	62 (16.26%)	87 (19.86%)	59 (25.00%)	22 (17.19%)	230	19.48%
	>8.5 cc	64 (16.89%)	103 (23.52%)	48 (20.34%)	29 (22.66%)	244	20.66%
Histology	Breast	65 (17.15%)	92 (21.00%)	59 (25.00%)	43 (33.59%)	259	21.93%
	GI	42 (11.08%)	38 (8.68%)	11 (4.66%)	1 (0.78%)	92	7.79%
	Gynae	11 (2.90%)	8 (1.83%)	9 (3.81%)	0 (0%)	28	2.37%
	Lung	144 (37.99%)	202 (46.12%)	109 (46.19%)	56 (43.75%)	511	43.27%
	Melanoma	59 (15.57%)	57 (13.01%)	30 (12.71%)	17 (13.28%)	163	13.80%
	Other*	24 (6.33%)	14 (3.20%)	6 (2.54%)	4 (3.13%)	48	4.06%
	Renal	34 (8.97%)	27 (6.16%)	12 (5.08%)	7 (5.47%)	80	6.77%
Molecular subtype	None	288 (75.99%)	319 (72.83%)	161 (68.22%)	65 (50.78%)	833	70.53%
	Driver mutation	91 (24.01%)	119 (27.17%)	75 (31.78%)	63 (49.22%)	348	29.47%
	ALK	5 (5.49%)	10 (8.33%)	7 (9.33%)	9 (14.29%)	31	2.62%
	EGFR	13 (14.29%)	34 (28.33%)	18 (24.00%)	14 (22.22%)	79	6.69%
	BRAF	32 (35.16%)	29 (24.17%)	18 (24.00%)	12 (19.05%)	91	7.71%
	HER2	41 (45.05%)	47 (29.17%	32 (42.67%)	28 (44.44%)	148	12.53%

Other* includes Urological Cancer, Cancer of Unknown Primary, Sarcoma, Thyroid Cancer and Head and Neck Cancer.

**Table 2:** Median survival according to primary histology, with breast cancer taken as the reference group.

Group	N	Median Survival	Hazard Ratio
Breast (ref)	259	14.76	–
Melanoma	163	17.59 (*p* = 0.2451)	0.87 (0.69–1.10)
Lung	511	9.90 (*p* = 0.0021)	1.32 (1.11–1.56)
GI	92	5.13 (*p* ≤ 0.0001)	2.51 (1.80–3.50)
Renal	80	10.22 (*p* = 0.2129)	1.20 (0.88–1.64)
Gynae	28	8.62 (*p* = 0.0854)	1.43 (0.89–2.30)
Other*	48	12.00 (*p* = 0.5625)	1.11 (0.77–1.60)

Other* includes Urological Cancer, Cancer of Unknown Primary, Sarcoma, Thyroid Cancer and Head and Neck Cancer.

## Data Availability

The data supporting these results are stored securely on the The Royal Marsden Hospital network servers. Please contact authors for access.
